# ﻿Identification of three novel species and one new record of *Kirschsteiniothelia* (Kirschsteiniotheliaceae, Kirschsteiniotheliales) from Jiangxi, China

**DOI:** 10.3897/mycokeys.112.142028

**Published:** 2025-01-23

**Authors:** Xing-Xing Luo, Ming-Gen Liao, Ya-Fen Hu, Xiu-Guo Zhang, Zhao-Huan Xu, Jian Ma

**Affiliations:** 1 College of Agronomy, Jiangxi Agricultural University, Nanchang, Jiangxi 330045, China; 2 Shandong Provincial Key Laboratory for Biology of Vegetable Diseases and Insect Pests, College of Plant Protection, Shandong Agricultural University, Taian, Shandong 271018, China; 3 Jiangxi Key Laboratory for Excavation and Utilization of Agricultural Microorganisms, Jiangxi Agricultural University, Nanchang, Jiangxi 330045, China

**Keywords:** Dothideomycetes, new species, phylogeny, saprobic fungi, taxonomy

## Abstract

Fungal diversity is rapidly expanding, with numerous species being discovered worldwide. While plant debris is a habitat favoring the survival and multiplication of various microbial species. In this study, several kirschsteiniothelia-like isolates were collected from dead branches of unidentified perennial dicotyledonous plants. Based on morphological examination and phylogenetic analyses of combined ITS, LSU, and SSU sequences data using maximum-likelihood and Bayesian inference, three new species of *Kirschsteiniothelia*, namely *K.ganzhouensis*, *K.jiangxiensis*, and *K.jiulianshanensis*, were introduced, and one known species, *K.inthanonensis*, was recorded for the first time from China. To improve our comprehensive knowledge of the species diversity of *Kirschsteiniothelia*, all accepted *Kirschsteiniothelia* species with morphological characteristics, sequence data, asexual morphs, habitat, host, and locality are listed.

## ﻿Introduction

Fungi are a diverse group of organisms that widely exist in nature and play an important role in ecosystem processes and functioning (Schimann et al. 2017). To date, approximately 165,000 fungal species have been recorded (Hyde 2022; Phukhamsakda et al. 2022; Index Fungorum 2024), but this is only a tiny fraction of the 2 to 11 million estimated species (Phukhamsakda et al. 2022; Niskanen et al. 2023), and many hidden species are still waiting to be explored. In recent years, the fungal diversity in China has drawn the attention of taxonomists, and a total of 27,807 fungal species and subspecies have been recorded by the Catalogue of Life China 2024 Annual Checklist. The recorded database strongly suggests that more research on fungal diversity in China is needed.

The genus *Kirschsteiniothelia* D. Hawksw. was introduced by Hawksworth (1985) for six combinations derived from *Microthelia* Körb. [= *Anisomeridium* (Müll. Arg.) M. Choisy] and *Sphaeria* Haller (= *Hypoxylon* Bull.) and was mainly characterized by superficial to semi-immersed, globose or subglobose, dark brown to black ascomata with fissitunicate, cylindrical or clavate, bitunicate, 8-spored asci and brown to dark brown, ellipsoidal, smooth-walled, 1(–2)-septate ascospores with or without a mucilaginous sheath (Hawksworth 1985; Boonmee et al. 2012; Hyde et al. 2013, Mehrabi et al. 2017). The generic type species, *K.aethiops* (Sacc.) D. Hawksw., has been linked with the asexual fungus *Dendryphiopsisatra* (Corda) S. Hughes (generic type) based on pure culture and sequence data (Hughes 1978; Hawksworth 1985; Boonmee et al. 2012). Wijayawardene et al. (2014) further proposed to use the name *Kirschsteiniothelia* over *Dendryphiopsis* S. Hughes, considering the requirement for fewer name changes, and made the correct name *Kirschsteiniotheliaatra* (Corda) D. Hawksw. [≡ *Dendryphiopsisatra* (Corda) S. Hughes] as the type species. Su et al. (2016) first reported the sporidesmium-like asexual morph (*K.submersa* Hong Y. Su & K.D. Hyde) in *Kirschsteiniothelia* based on molecular evidence, and later the sporidesmium-like asexual morphs were frequently reported in *Kirschsteiniothelia* with undetermined sexual morphs (Li et al. 2016; Hyde et al. 2017; Bao et al. 2018; Sun et al. 2021; Jayawardena et al. 2022; Hyde et al. 2023; Liu et al. 2023; Xu et al. 2023; Yang et al. 2023; Zhang et al. 2023; de Farias et al. 2024; Sruthi et al. 2024). Thus, *Kirschsteiniothelia* has two types of asexual morphs, namely dendryphiopsis-like and sporidesmium-like. The dendryphiopsis-like asexual morph is characterized by macronematous, branched at the apex, forming a stipe and head, brown to dark brown, determinate or percurrently extending conidiophores with mono- to polytretic, integrated, terminal and lateral conidiogenous cells that produce acrogenous, solitary or catenate, septate conidia. The sporidesmium-like asexual morph has macronematous, unbranched conidiophores with integrated, terminal, monoblastic or monotretic, determinate or irregularly extending conidiogenous cells that produce acrogenous, solitary or catenate, septate conidia with or without a mucilaginous sheath (Sun et al. 2021; Liu et al. 2023; Xu et al. 2023; Sruthi et al. 2024; Tang et al. 2024).

*Kirschsteiniothelia* is a genus of the Dothideomycetes O.E. Erikss. & Winka, of which familial placements have undergone several revisions. It was originally assigned to the family Pleosporaceae Nitschke by Hawksworth (1985) and later transferred to Pleomassariaceae M.E. Barr by Barr (1993) based on host, morphology, and mimicry. Schoch et al. (2006) revealed that *K.aethiops* (generic type) was not phylogenetically close to Pleosporaceae based on molecular data and should be placed in a separate family. Schoch et al. (2009) and Suetrong et al. (2009) further showed that two other *Kirschsteiniothelia* species, *K.elaterascus* Shearer and *K.maritima* (Linder) D. Hawksw., clustered into Morosphaeriaceae Suetrong, Sakay., E.B.G. Jones & C.L. Schoch and Mytilinidiaceae Kirschst., respectively. On this basis, Boonmee et al. (2012) introduced a new family, Kirschsteiniotheliaceae Boonmee & K.D. Hyde, to accommodate taxa grouping with *K.aethiops* based on combined ITS, LSU, and SSU sequence data, and transferred *K.elaterascus* and *K.maritima* to *Morosphaeria S*uetrong, Sakay., E.B.G. Jones & C.L. Schoch and *Halokirschsteiniothelia* Boonmee & K.D. Hyde, respectively. Later, Hernandez-Restrepo et al. (2017) treated Kirschsteiniotheliaceae in a new order, Kirschsteiniotheliales Hern.-Restr., R.F. Castañeda, Gené & Crous, based on its distant relationship to other lineage representatives of different orders in Dothideomycetes. Hongsanan et al. (2020) further showed that Kirschsteiniotheliales cluster with Asterinales M.E. Barr ex D. Hawksw. & O.E. Erikss., but diverged around 221 MYA.

Jiangxi Province is located in the southeast of China. Its rich vegetation and subtropical climatic regimes favor the survival and multiplication of various microbial species. However, its mycobiota, especially of mitosporic fungi, is relatively backward. During our continuing survey of saprophytic microfungi from this region, several interesting hyphomycetes were collected on dead branches of unidentified plants. Both molecular analyses and morphological data placed four species within the genus *Kirschsteiniothelia*. Three of these, namely *K.ganzhouensis*, *K.jiangxiensis*, and *K.jiulianshanensis*, are introduced as new to science, while the fourth is *K.inthanonensis* J. Louangphan & Gomes de Farias, a new record from China.

## ﻿Materials and methods

### ﻿Collections and examination of specimens

The samples of dead branches were collected randomly from the forest ecosystem of Guanshan and Jiulianshan National Nature Reserves, Jiangxi Province, China, placed in Ziplock plastic bags with collection information (Rathnayaka et al. 2024), and taken to the laboratory of conservation and utilization of fungal resources. Samples were processed and examined following the methods described in Ma et al. (2011). Colonies present on the surface of dead branches were examined and observed visually using a stereomicroscope (Motic SMZ-168, Xiamen, China) at varying magnifications ranging from 0.75 to 5 times. Fresh colonies were isolated with a sterile needle at 5 × magnification under a stereomicroscope, mounted on a slide with a drop of lactic acid-phenol solution (lactic acid, phenol, glycerol, and sterile water in proportions of 1:1:2:1), and subsequently scrutinized under an Olympus BX 53 light microscope equipped with an Olympus DP 27 digital camera (Olympus Optical Co., Tokyo, Japan) for microscopic morphological characterization. The conidia of the target colony were directly collected from the specimen using the tip of a sterile toothpick dipped in 40% sterile glycerin water. These conidia were then placed on the surface of PDA (20% potato + 2% dextrose + 2% agar, wt/vol) and incubated at 25 °C. The individual germinated conidia were transferred to fresh PDA plates and incubated in an incubator maintained in darkness at 25 °C. Culture characteristics were meticulously examined and recorded after 5 days. Colony colors were evaluated according to Rayner’s charts (Rayner 1970). All fungal strains have been preserved in sterilized glycerin at a concentration of 10% and stored at temperatures of approximately 4 °C for future studies. The specimens and cultures studied have been archived within the Herbarium of Jiangxi Agricultural University, Plant Pathology, Nanchang, China (HJAUP). The names assigned to new taxa have been officially registered within MycoBank (http://www.mycobank.org).

### ﻿DNA extraction, PCR amplification, and sequencing

Total genomic DNA was extracted from fungal cultures grown on PDA plates for 2 weeks at 25 °C using the Solarbio Fungal Genomic DNA Extraction Kit (Beijing Solarbio Science & Technology Co., Ltd., Beijing, China). Three different gene regions, ITS, LSU, and SSU, were selected for this study. Primer pairs ITS5/ITS4 (White et al. 1990), 28S1-F/28S3-R, and 18S-F/18S-R (Xia et al. 2017) were used to amplify parts of the ITS, LSU, and SSU loci, respectively. The final volume of the PCR reaction was carried out in a 20 μL reaction volume containing 10 μL of 2 × Power Taq PCR MasterMix, 0.8 μL each of forward and reverse primer, 1 μL of DNA template, and 7.4 µL of ddH_2_O. The PCR thermal cycling conditions of ITS, LSU, and SSU were initialized at 94 °C for 3 min, followed by 35 cycles of denaturation at 94 °C for 15 s, annealing at 54 °C for 15 s, elongation at 72 °C for 30 s, a final extension at 72 °C for 10 min, and finally kept at 4 °C. The PCR products were visualized on 1% agarose gel electrophoresis stained with ethidium bromide. Sequencing was performed bidirectionally by Hunan Youkanglai Biotechnology Co., Ltd., Changsha, China. Newly obtained sequences in this study have been deposited in NCBI GenBank (www.ncbi.nlm.nih.gov, accessed on 25 May 2024; Table 1).

**Table 1.** Names, strain numbers, and corresponding GenBank accessions of *Kirschsteiniothelia* taxa used in the phylogenetic analyses. New sequences are indicated in bold.

Species	Strain Number	GenBank Accession Numbers		
		ITS	LSU	SSU
*Acrospermumadeanum*	M133	EU940180	EU940104	EU940031
*A.compressum*	M151	EU940161	EU940084	EU940012
*A.gramineum*	M152	EU940162	EU940085	EU940013
*Anisomeridiumubianum*	MPN94	–	GU327709	JN887379
*Flavobatheliumepiphyllum*	MPN67	–	GU327717	JN887382
*Kirschsteiniotheliaacutispora*	MFLU 21-0127	OP120780	ON980758	ON980754
*K.agumbensis*	NFCCI 5714 ^T^	PP029048	–	PP029049
*K.aquatica*	MFLUCC 17-1685 ^T^	MH182587	MH182594	MH182618
*K.arasbaranica*	IRAN 2509C	KX621986	KX621987	KX621988
*K.arasbaranica*	IRAN 2508C ^T^	KX621983	KX621984	KX621985
*K.atra*	CBS 109.53	–	AY016361	AY016344
*K.atra*	MFLUCC 15-0424	KU500571	KU500578	KU500585
*K.bulbosapicalis*	GZCC 23-0732 ^T^	PQ248937	PQ248933	PQ248929
*K.cangshanensis*	MFLUCC 16-1350 ^T^	MH182584	MH182592	–
*K.chiangmaiensis*	MFLU 23-0358 ^T^	OR575473	OR575474	OR575475
*K.crustacea*	MFLU 21-0129 ^T^	MW851849	MW851854	–
*K.dendryphioides*	KUNCC 10431 ^T^	OP626354	PQ248935	PQ248931
*K.dendryphioides*	KUNCC 10499	PQ248938	–	–
*K.dushanensis*	18D-43 ^T^	OP377845	–	–
*K.ebriosa*	CBS H-23379	–	LT985885	–
*K.emarceis*	MFLUCC 10-0037 ^T^	HQ441570	HQ441571	HQ441572
*K.esperanzae*	T. Raymundo 6581 ^T^	OQ877253	OQ880482	–
*K.extensa*	MFLU 21-0130 ^T^	MW851850	MW851855	–
*K.fluminicola*	MFLUCC 16-1263 ^T^	MH182582	MH182588	–
***K.ganzhouensis***	**HJAUP C1209 ^T^**	**PP505546**	**PP506568**	**PP527763**
***K.ganzhouensis***	**HJAUP C1210**	**PQ456024**	**PQ443751**	**PQ443763**
***K.ganzhouensis***	**HJAUP C1211**	**PQ456025**	**PQ443752**	**PQ443764**
*K.guangdongensis*	ZHKUCC 22-0233 ^T^	–	OR164974	–
*K.inthanonensis*	MFLUCC 23–0277 ^T^	OR762773	OR762781	OR764784
***K.inthanonensis***	**HJAUP C1502**	**PQ456029**	**PQ443756**	**PQ443768**
***K.inthanonensis***	**HJAUP C1503**	**PQ456030**	**PQ443757**	**PQ443769**
***K.jiangxiensis***	**HJAUP C1273 ^T^**	**PP505548**	**PP506566**	**PP506565**
***K.jiangxiensis***	**HJAUP C1274**	**PQ456026**	**PQ443753**	**PQ443765**
***K.jiangxiensis***	**HJAUP C1275**	**PQ456027**	**PQ443754**	**PQ443766**
***K.jiulianshanensis***	**HJAUP C1313 ^T^**	**PP505549**	**PP506562**	**PP506563**
***K.jiulianshanensis***	**HJAUP C1314**	**PQ456028**	**PQ443755**	**PQ443767**
*K.laojunensis*	KUN L88727 ^T^	PP081658	–	PP081651
*K.lignicola*	MFLUCC 10-0036 ^T^	HQ441567	HQ441568	HQ441569
*K.longirostrata*	GZCC 23-0733 ^T^	PQ248939	PQ248934	PQ248930
*K.longisporum*	UESTCC 24.0190 ^T^	PQ038266	PQ038273	PQ046108
*K.nabanheensis*	HJAUP C2004 ^T^	OQ023197	OQ023273	OQ023038
*K.nabanheensis*	HJAUP C2006	OQ023274	OQ023275	OQ023037
*K.phoenicis*	MFLUCC 18-0216 ^T^	MG859978	MG860484	MG859979
*K.pini*	UESTCC 24.0131 ^T^	**PP835321**	**PP835315**	**PP835318**
*K.puerensis*	ZHKUCC 22-0271 ^T^	OP450977	OP451017	OP451020
*K.puerensis*	ZHKUCC:22-0272	OP450978	OP451018	OP451021
*K.ramus*	GZCC:23-0596 ^T^	OR098711	OR091333	–
*K.rostrata*	MFLUCC 15-0619 ^T^	KY697280	KY697276	KY697278
*K.rostrata*	MFLUCC 16-1124	–	MH182590	–
*K.saprophytica*	MFLUCC 23–0275 ^T^	OR762774	OR762783	–
*K.saprophytica*	MFLUCC 23–0276	OR762775	OR762782	–
*K.septemseptata*	MFLU 21-0126 ^T^	OP120779	ON980757	ON980752
*K.sichuanensis*	UESTCC 24.0127 ^T^	PP785368	PP784322	–
*K.spatiosa*	MFLU 21-0128 ^T^	OP077294	–	ON980753
*K.submersa*	MFLUCC 15-0427 ^T^	KU500570	KU500577	KU500584
*K.submersa*	S-481	–	MH182591	MH182616
*K.tectonae*	MFLUCC 12-0050 ^T^	KU144916	KU764707	–
*K.tectonae*	MFLUCC 23-0272	OR762772	OR762780	OR764783
*K.thailandica*	MFLUCC 20-0116 ^T^	MT985633	MT984443	MT984280
*K.thujina*	JF 13210 ^T^	KM982716	KM982718	KM982717
*K.vinigena*	CBS H-23378 ^T^	–	LT985883	–
*K.xishuangbannaensis*	ZHKUCC 22-0220 ^T^	OP289566	OP303181	OP289564
*K.xishuangbannaensis*	ZHKUCC 22-0221	OP289563	OP303182	OP289565
*K.zizyphifolii*	MFLUCC 23–0270 ^T^	OR762768	OR762776	OR764779
*Megalotremisverrucosa*	MPN104	–	GU327718	JN887383
*Phyllobatheliumanomalum*	MPN 242	–	GU327722	JN887386
*P.firmum*	ERP 3175	–	GU327723	–
*Pseudorobillardaeucalypti*	MFLUCC 12-0422	KF827451	KF827457	KF827463
*Ps.phragmitis*	CBS 398.61	MH858101	EU754203	EU754104
*Strigulaguangxiensis*	HMAS-L0138040 ^T^	KY100301	MK206256	–
*S.nemathora*	MPN 72	–	JN887405	JN887389
*Tenuitholiascusporinoides*	HMAS-L0139638 ^T^	–	MK206259	MK352441
*T.porinoides*	HMAS-L0139639	–	MK206258	MK352442
*T.porinoides*	HMAS-L0139640	–	MK206260	MK352443

Notes: “^T^” indicates ex-type strain. “—” stands for unavailability of sequence data in GenBank.

### ﻿Phylogenetic analyses

Novel sequences were generated from ten strains in this study, and all available reference sequences of *Kirschsteiniothelia* species were downloaded from GenBank. All sequences in this study included in the phylogenetic analyses are summarized in Table 1. Each gene region was independently aligned using the online version of MAFFT v.7 (Katoh and Standley 2013) on the web server (http://maffth.cbrc.jp/alignment/server/, accessed on 10 December 2024). The alignment was reviewed in MEGA v.7, followed by minor manual adjustments to ensure character homology between taxa. A matrix was formed with 60 strains (514 characters) for ITS, 70 strains (581 characters) for LSU, and 55 strains (1,239 characters) for SSU. The aligned matrices were concatenated into a single matrix (74 strains, 2334 characters). These sequence data were concatenated by Phylosuite software v1.2.1 within “Concatenate Sequence” (Zhang et al. 2020), and the concatenated aligned dataset was analyzed separately using maximum-likelihood (ML) and Bayesian inference (BI). The best evolutionary model for each alignment dataset was constructed using ModelFinder (Kalyaanamoorthy et al. 2017). Maximum-likelihood phylogenies were inferred using IQ-TREE (Nguyen et al. 2015) within 10,000 ultrafast bootstraps (Minh et al. 2013) under the best partitioned model. The optima trees were inferred using the heuristic search option with 1000 random sequence additions. The best-fit model was TN+F+I+G4 for ITS and LSU and TNe+I+G4 for SSU alignments. Bayesian inference phylogenies were inferred using MrBayes 3.2.6 (Ronquist et al. 2012) based on the partition model (2 parallel runs, 2,000,000 generations), and the best nucleotide substitution model for each locus was identified using ModelFinder of Phylosuite software v1.2.1 to be GTR+F+I+G4 for ITS and LSU and SYM+I+G4 for SSU. The resulting trees were visualized using FigTree v.1.4.2 (Zhang et al. 2020) (http://tree.bio.ed.ac.uk/software/figtree, accessed on 10 December 2024) and further edited in Adobe Illustrator 2021. The alignments and trees were deposited in TreeBASE: S31882 (http://treebase.org/treebase-web/home.html).

## ﻿Results

### ﻿Molecular phylogeny

**Figure 1.**
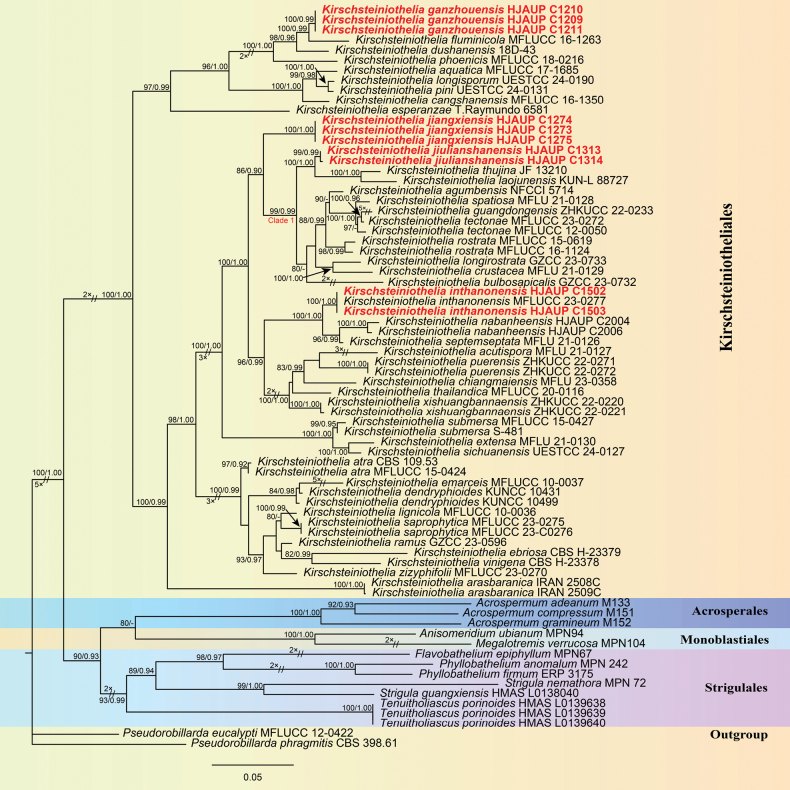
Maximum-likelihood phylogenetic tree of *Kirschsteiniothelia* based on the combined ITS, LSU, and SSU sequence data. The ML and BI bootstrap support values equal to or above 80% and 0.90 are given above the nodes. Bar = 0.06 substitutions per nucleotide position. The tree was rooted to *Pseudorobillardaeucalypti* (MFLUCC 12-0422) and *Ps.Phragmitis* (CBS 398.61). Strains of the species from the present study were marked in red. Orders were indicated on the right side of the tree in blocks. Some branches are shortened according to the indicated multipliers to fit the page size, and these are indicated by the symbol (//).

The phylogenetic tree inferred from maximum-likelihood and Bayesian inference analyses based on combined ITS, LSU, and SSU sequence data consisted of four orders (Acrosperales, Kirschsteiniotheliales, Monoblastiales, and Strigulales). The concatenated sequence matrix comprised 74 sequences with 2334 total characters in the combined dataset (ITS: 1–514, LSU: 515–1095, SSU: 1096–2334), 1151 distinct patterns, 670 parsimony informative sites (ITS: 270, LSU: 237, SSU: 163), 349 singleton sites, and 1315 constant sites. *Pseudorobillardaeucalypti* (MFLUCC 12-0422) and *Ps.phragmitis* (CBS 398.61) were regarded as the outgroup. The phylogenetic trees have a similar topology, obtained from the combined dataset of maximum-likelihood and Bayesian inference analyses. The best-scoring ML concatenated tree (lnL = –18756.227) with superimposed posterior probabilities from MrBayes analysis is shown in Fig. 1. Phylogenetic analyses of the ITS+LSU+SSU concatenated datasets showed that these ten strains nested within the genus *Kirschsteiniothelia*, representing four independent lineages (Fig. 1). *Kirschsteiniotheliaganzhouensis* (HJAUP C1209, HJAUP C1210, and HJAUP C1211) clustered sister to *K.fluminicola* (MFLUCC 16–1263) with 100% ML/0.99 BI bootstrap support. *Kirschsteiniotheliajiangxiensis* (HJAUP C1273, HJAUP C1274, and HJAUP C1275) formed an independent lineage basal to Clade 1 with 86% ML/0.90 BI bootstrap support. *Kirschsteiniotheliajiulianshanensis* (HJAUP C1313 and HJAUP C1314) forms a distinct clade sister to the clade containing *K.thujina* (JF 13210) and *K.laojunensis* (KUN-L 88727) with 100% ML/1.00 BI bootstrap support. In addition, our new collection (HJAUP C1502 and HJAUP C1503) clustered together with the known species *K.inthanonensis* (MFLUCC 23-0277) with 100% ML/1.00 BI bootstrap support, indicating they represent the same species.

### ﻿Taxonomy

#### ﻿ Kirschsteiniothelia ganzhouensis

Taxon classificationFungiKirschsteiniothelialesKirschsteiniotheliaceae

Y.F. Hu & Jian Ma sp. nov.

48F2DAB9-0CCA-55D9-A8F0-12DB909414E5

856638

Fig. 2

##### Type.

China • Jiangxi Province, Ganzhou City, Longnan County, Jiulianshan National Nature Reserve, on dead branches of an unidentified broadleaf tree, 29 June 2022, Y.F. Hu (HJAUP M1209, *holotype*), ex-type living culture, HJAUP C1209 = HJAUP C1210 = HJAUP C1211.

##### Etymology.

The name refers to the type locality “Ganzhou City”.

##### Description.

Saprobic on decaying wood in terrestrial habitats. Asexual morph: Hyphomycetes. Colonies on natural substratum effuse, dark brown, hairy. Mycelium superficial and immersed, composed of branched, dark brown to black, septate, smooth-walled hyphae. Conidiophores macronematous, mononematous, erect, straight or flexuous, irregular or subscorpioid branched near the apex, cylindrical, smooth, septate, dark brown to black, 146.8–200 × 7.1–10.1 μm (x̄ = 176.1 × 8.0 μm, SD = 21 × 1, n = 15). Conidiogenous cells monotretic, integrated, terminal or intercalary, cylindrical, pale brown to brown, determinate, or sometimes with several cylindrical, enteroblastic percurrent extensions. Conidia acrogenous, solitary, obclavate, straight or slightly curved, sometimes rostrate, smooth, subhyaline to pale brown, 2–7(–14)-distoseptate, 20.3–65.8(–164) × 3.0–5.3 μm (x̄ = 36.4 × 4.7 μm, SD = 12 × 0.36, n = 20), tapering to 1.3–2.6 μm near the apex, 3.0–5.3 μm wide at the base, and rounded at the apex. Sexual morph: Undetermined.

**Figure 2.**
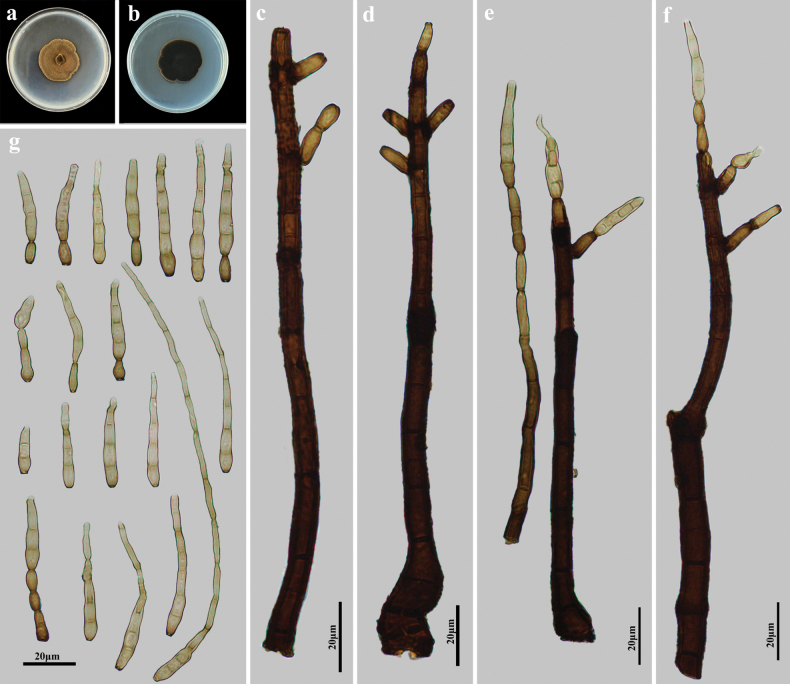
*Kirschsteiniotheliaganzhouensis* (HJAUP M1209, holotype) **a**, **b** colonies after 4 weeks on PDA (front and reverse) **c**, **d** conidiophores and conidiogenous cells **e**, **f** conidiophores, conidiogenous cells, and conidia **g** conidia.

##### Culture characteristics.

Colonies growing on PDA medium reaching 30–35 mm diam. after 4 weeks at 25 °C in darkness, irregular circular, surface yellow-brown with fluffy hyphae, reverse dark brown to black.

##### Note.

The phylogenetic tree showed that *K.ganzhouensis* (HJAUP C1209, HJAUP C1210, and HJAUP C1211) clusters with *K.fluminicola* (MFLUCC 16-1263). Based on the BLASTn results, ITS and LSU gene sequences of *K.ganzhouensis* (HJAUP C1209) showed 93% (484/520, 3 gaps) and 99% (518/525, 0 gap) similarities to *K.fluminicola* (MFLUCC 16-1263), respectively. Moreover, *K.ganzhouensis* differs morphologically from *K.fluminicola* Z.L. Luo, K.D. Hyde & H.Y. Su (Bao et al. 2018) in having monotretic conidiogenous cells, shorter conidiophores (146.8–200 μm vs. 209–286 μm), and smaller conidia (20.3–65.8 × 3.0–5.3 μm vs. 47.5–86.5 × 8–10 μm). In addition, *K.ganzhouensis* further differs from *K.fluminicola* in that it occurs in a terrestrial habitat and not in a freshwater habitat.

#### ﻿ Kirschsteiniothelia inthanonensis

Taxon classificationFungiKirschsteiniothelialesKirschsteiniotheliaceae

J. Louangphan & Gomes de Farias, 2024

B1EFEF3C-C545-5F31-B212-C4F58C9C0BF3

Index Fungorum: IF901384

Facesoffungi Number: FoF14982

Fig. 3

##### Description.

Saprobic on decaying wood in terrestrial habitats. Asexual morph: Hyphomycetes. Colonies on natural substratum effuse, dark brown, hairy. Mycelium immersed and superfcial, composed of branched, septate, dark brown to black, smooth-walled hyphae. Conidiomata synnematous, solitary, erect, cylindrical, dark brown to black, becoming narrower toward the apex, up to 1266 μm high, 110–330 μm wide at the swollen base. Conidiophores distinct, macronematous, erect, straight or flexuous, closely fasciculate, branched near the apex, septate, smooth, cylindrical, brown to dark brown, up to 1266 μm long, 4.8–8 μm wide, diverging laterally and terminally. Conidiogenous cells monotretic, integrated, terminal, cylindrical, smooth, brown, determinate, or sometimes with several cylindrical, enteroblastic percurrent extensions. Conidia acrogenous, solitary or catenate, obclavate, straight or slightly curved, smooth, olivaceous brown to brown, 2–5-euseptate, 20–48 × 8–13.3 μm (x̄ = 31.4 × 9.8 μm, SD = 9 × 1, n = 30), partly tapering towards and rounded at the apex. Sexual morph: Undetermined.

**Figure 3.**
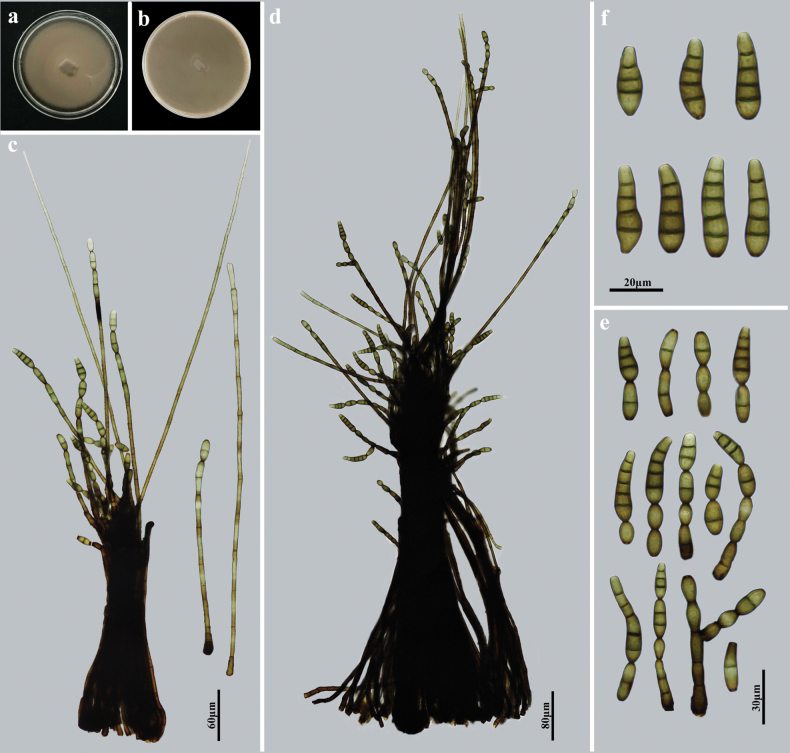
*Kirschsteiniotheliainthanonensis* (HJAUP M1502, holotype) **a**, **b** colonies after 4 weeks on PDA (front and reverse) **c**, **d** synnemata with conidiophores, conidiogenous cells, and conidia **e** conidiogenous cells and conidia **f** conidia.

##### Culture characteristics.

Colonies growing on PDA medium reaching 85–90 mm diam. after 4 weeks at 25 °C in darkness, circular, surface velvety, with reddish-brown to brown mycelium, reverse brown to dark brown.

##### Material examined.

China • Jiangxi Province, Ganzhou City, Longnan County, Jiulianshan Town, Guanshan National Nature Reserve, on dead branches of an unidentified broadleaf tree, 27 June 2021, Y.F. Hu (HJAUP M1502, *holotype*), living culture, HJAUP C1502 = HJAUP C1503.

##### Note.

*Kirschsteiniotheliainthanonensis* was originally described with an asexual morph on the twigs of *Quercusoleoides* in Thailand (de Farias et al. 2024) and was known only from its type collection. Morphologically, our new collection shows high morphological similarity to *K.inthanonensis* except for its wider conidiophores (4.8–8 μm vs. 2.5–6.6 μm), shorter conidia (20–48 μm vs. 24–230 μm) with fewer septa (2–5-euseptate vs. 2–10-euseptate) (de Farias et al. 2024). In addition, the phylogenetic tree showed that our new collection (HJAUP C1502 and HJAUP C1503) clustered with *K.inthanonensis* (MFLUCC 23-0277). Based on pairwise nucleotide comparisons of ITS, LSU, and SSU, their nucleotide differences (0/517 in ITS, 2/565 in LSU, and 0/1022 in SSU) are minor. Therefore, we identified our new collection as *K.inthanonensis*, and it is a new record for China.

#### ﻿ Kirschsteiniothelia jiangxiensis

Taxon classificationFungiKirschsteiniothelialesKirschsteiniotheliaceae

Y.F. Hu & Jian Ma sp. nov.

720D2737-2DDF-5E9E-9C11-061DD25B64AD

856639

Fig. 4

##### Type.

China • Jiangxi Province, Ganzhou City, Longnan County, Jiulianshan National Nature Reserve, on dead branches of an unidentified broadleaf tree, 29 June 2022, Y.F. Hu (HJAUP M1273, *holotype*), ex-type living culture, HJAUP C1273 = HJAUP C1274 = HJAUP C1275.

##### Etymology.

The name refers to the locality “Jiangxi Province”, from where the fungus was collected.

##### Description.

Saprobic on decaying wood in terrestrial habitats. Asexual morph: Hyphomycetes. Colonies on natural substratum effuse, dark brown, hairy. Mycelium superficial and immersed, composed of branched, septate, dark brown to black, smooth-walled hyphae. Conidiophores macronematous, mononematous, simple or branched, erect, straight or flexuous, cylindrical, smooth, septate, dark brown to black, 32.9–90.4 × 7.3–12.9 μm (x̄ = 48.7 × 9.1 μm, SD = 17 × 2, n = 15). Conidiogenous cells monoblastic, integrated, terminal, cylindrical, smooth, brown to dark brown, determinate, or sometimes with several cylindrical, enteroblastic percurrent extensions. Conidia solitary, acrogenous, obclavate, straight or curved, smooth, brown, 7–10-euseptate, 75.9–103.8 × 8.9–15.2 μm (x̄ = 90.8 × 10.9 μm, SD = 8 × 2, n = 20), tapering to 2.5–5.6 μm at the apex, 5.3–7.6 μm wide at the truncate base, and rounded at the apex. Sexual morph: Undetermined.

**Figure 4.**
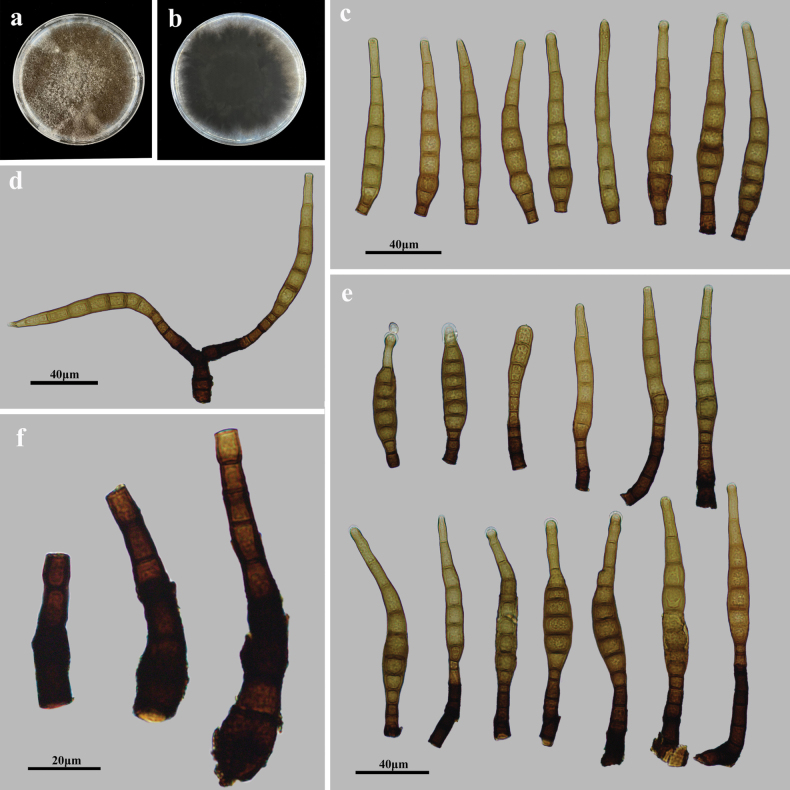
*Kirschsteiniotheliajiangxiensis* (HJAUP M1273, holotype) **a**, **b** colonies after 4 weeks on PDA (front and reverse) **c** conidia **d**, **e** conidiophores, conidiogenous cells, and conidia **f** conidiophores and conidiogenous cells.

##### Culture characteristics.

Colonies growing on PDA medium reaching 85–90 mm diam. after 4 weeks at 25 °C in darkness, irregular circular, surface velvety, grey-white in center and brown at margin with dense mycelium, reverse dark brown to black.

##### Note.

The phylogenetic tree showed that *K.jiangxiensis* (HJAUP C1273, HJAUP C1274, and HJAUP C1275) belongs to *Kirschsteiniothelia* and forms a distinct lineage sister to Clade 1. However, *K.jiangxiensis* (HJAUP C1273) differs from the morphologically most similar species, *K.spatiosa* (MFLU 21-0128) (Jayawardena et al. 2022), in having shorter conidiophores [32.9–90.4 μm (x̄ = 48.7) vs. 70–128 µm (x̄ = 100)] and smaller conidia [75.9–103.8 × 8.9–15.2 μm (x̄ = 90.8 × 10.9 μm) vs. 90–139 μm × 9.5–16.5 µm (x̄ = 113 × 14 μm)] with fewer septa (7–10 vs. 8–23), and further from *K.spatiosa* by 96 nucleotides (67/380 in ITS and 26/1032 in SSU). In addition, *K.jiangxiensis* also differs from other taxa in Clade 1 in the size of conidiophores and conidia.

#### ﻿ Kirschsteiniothelia jiulianshanensis

Taxon classificationFungiKirschsteiniothelialesKirschsteiniotheliaceae

Y.F. Hu & Jian Ma sp. nov.

4CE278A2-14E1-573E-98C3-0C4A37675D4E

856640

Fig. 5

##### Type.

China • Jiangxi Province, Ganzhou City, Longnan County, Jiulianshan National Nature Reserve, on dead branches of an unidentified broadleaf tree, 29 June 2022, Y.F. Hu (HJAUP M1313, *holotype*), ex-type living culture, HJAUP C1313 = HJAUP C1314.

##### Etymology.

The name refers to Jiulianshan National Nature Reserve, the locality where the fungus was collected.

##### Description.

Saprobic on decaying wood in terrestrial habitats. Asexual morph: Hyphomycetes. Colonies on natural substratum effuse, dark brown, hairy. Mycelium immersed and superficial, composed of branched, dark brown to black, septate, smooth-walled hyphae. Conidiophores macronematous, mononematous, unbranched, erect, straight or flexuous, cylindrical, smooth, dark brown to black, 7–17-septate, 128.6–291.4(–430) × 7.1–10 μm (x̄ = 217.4 × 8.46 μm, SD = 85 × 0.7, n = 15). Conidiogenous cells monotretic, integrated, terminal, cylindrical, brown, determinate, or sometimes with several cylindrical, enteroblastic percurrent extensions. Conidia acrogenous, solitary, obclavate, straight or slightly curved, pale brown to brown, 4–7-euseptate, 31.4–57.1 × 10–11.4 μm (x̄ = 41.6 × 11.04 μm, SD = 9 × 0.5, n = 20), tapering to 4.6–6 μm at the apex, 2.3–3.4 μm wide at the truncate base, and rounded at the apex. Sexual morph: Undetermined.

**Figure 5.**
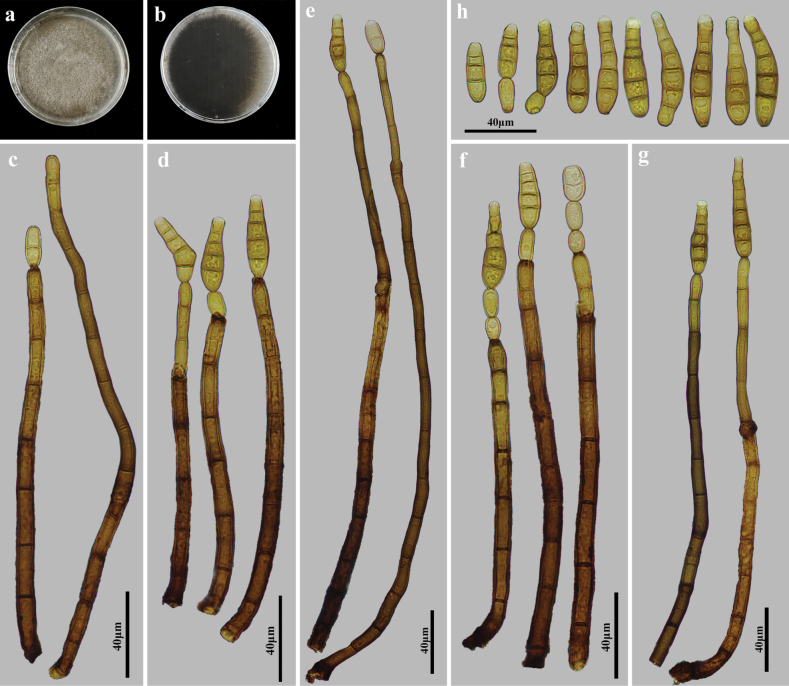
*Kirschsteiniotheliajiulianshanensis* (HJAUP M1313, holotype) **a, b** colonies after 4 weeks on PDA (front and reverse) **c–g** conidiophores, conidiogenous cells, and conidia **h** conidia.

##### Culture characteristics.

Colonies growing on PDA medium reaching 85–90 mm diam. after 4 weeks at 25 °C in darkness, circular, surface velvety, grey-white in center and brown at margin with dense mycelium, reverse dark brown to black.

##### Note.

The phylogenetic tree showed that *K.jiulianshanensis* (HJAUP C1313 and HJAUP C1314) clusters with *K.laojunensis* (KUN-L 88727) and *K.thujina* (JF 13210). Based on nucleotide comparisons, *K.jiulianshanensis* (HJAUP C1313) and *K.laojunensis* (KUN-L 88727) showed 63 bp differences (3%, including eight gaps) in ITS and SSU regions; *K.jiulianshanensis* (HJAUP C1313) and *K.thujina* (JF 13210) showed 75 bp differences (7%, including twelve gaps) in ITS, LSU, and SSU regions. Moreover, *K.jiulianshanensis* was found only in its asexual morph, while *K.laojunensis* Q.F. Meng & S.B. Fu (Meng et al. 2024) and *K.thujina* (Peck) D. Hawksw. (Hawksworth 1985) are known only as the sexual morph. In addition, *K.jiulianshanensis* (HJAUP C1313) can be distinguished from the morphologically most similar species, *K.crustacea* S. Wang, Q. Zhao & K.D. Hyde (Jayawardena et al. 2022), in having longer conidiophores (128.6–291.4 μm vs. 60–170 μm) and smaller conidia (31.4–57.1 × 10–11.4 μm vs. 45–75 × 10–18 μm), and further from *K.crustacea* by 90 nucleotides (79/491 in ITS and 11/545 in SSU).

## ﻿Discussion

Saprobic fungi are highly diverse in freshwater and terrestrial habitats, and a large number of novel taxa have been reported from many natural substrates such as submerged wood, dead branches, bark, culms, and leaves (Ellis 1971, 1976; Wu and Zhuang 2005; Hernandez-Restrepo et al. 2017; Luo et al. 2019; Hyde et al. 2023; Dissanayake et al. 2024; Tang et al. 2024). In our study, numerous hyphomycetes were collected on dead branches from terrestrial habitats in Jiangxi Province, China. Based on morphological characteristics and multi-locus (ITS, LSU, and SSU) phylogenetic analyses, three new species of *Kirschsteiniothelia*, *viz. K.ganzhouensis*, *K.jiangxiensis*, and *K.jiulianshanensis*, and one new Chinese record, *K.inthanonensis*, were identified, which contributed to our understanding of the species diversity of this genus.

*Kirschsteiniothelia* was established by Hawksworth (1985), with *K.aethiops* as the type species. To date, 62 epithets of *Kirschsteiniothelia* have been recorded (Index Fungorum 2024; Sruthi et al. 2024; Tian et al. 2024), but *K.elaterascus* and *K.maritima* were respectively transferred to *Neohelicascus* W. Dong, H. Zhang, K.D. Hyde & Doilom and *Halokirschsteiniothelia* Boonmee & K.D. Hyde based on phylogenetic analyses (Boonmee et al. 2012; Dong et al. 2020). Wijayawardene et al. (2014) further proposed to use *Kirschsteiniothelia* over *Dendryphiopsis* in the context of the one fungus, one name initiative and synonymized *K.aethiops* with *K.atra* (Corda) D. Hawksw. Accordingly, *K.incrustans*, derived from *Microtheliaincrustans* (Ellis & Everh.) Corlett & S. Hughes (a synonym of *K.aethiops*), was also treated as the synonym of *K.atra* (Wijayawardene et al. 2014; Mehrabi et al. 2017; Index Fungorum 2024; Jin et al. 2024). Mehrabi et al. (2017) provided an identification key to 20 well-documented *Kirschsteiniothelia* species and listed their principal synonyms. Sun et al. (2021) provided a synopsis of 35 *Kirschsteiniothelia* species with distribution, habitat, host, and morphology type of each species, but *K.elaterascus*, *K.incrustans*, and *K.maritima* were previously rejected out of *Kirschsteiniothelia*, and the other five species, *viz. K.arbuscula*, *K.binsarensis*, *K.biseptata*, *K.fascicularis*, and *K.goaensis* in *Kirschsteiniothelia*, were invalid (Turland et al. 2018: Art. F.5.1: no identifier number cited, and Art. 41.1: lacking a full and direct basionym reference) until Sruthi et al. (2024) legitimately placed them as five new combinations of *Kirschsteiniothelia*. Xu et al. (2023) summarized the morphological, host, and location information of 29 *Kirschsteiniothelia* species. Subsequently, Sruthi et al. (2024) listed 34 asexual morphs under *Kirschsteiniothelia*, and Tang et al. (2024) provided a checklist for 59 *Kirschsteiniothelia* species with their host, habitat, country, and reported morph, but *K.dujuanhuensis* was unpublished. Thus, *Kirschsteiniothelia* currently comprises 58 valid species. All species are known for their asexual or sexual morphs, and only five species, namely *K.atra*, *K.emarceis*, *K.lignicola*, *K.recessa*, and *K.saprophytica*, are known from both morphs.

Traditionally, *Kirschsteiniothelia* species have been characterized and identified based on morphological characteristics, but the lack of molecular data made it difficult to evaluate the phylogenetic relationships and taxonomic placements of some doubtful or morphological similarity species. With the development of molecular technology, multi-gene combined analysis has gradually occupied a dominant status in fungal taxonomy. To date, there are 39 *Kirschsteiniothelia* species with molecular data (Tang et al. 2024), and recent studies indicated that the concatenated dataset of ITS, LSU, and SSU sequences shows good resolution in revealing the phylogeny of *Kirschsteiniothelia*. Since 2012, all described *Kirschsteiniothelia* species were identified using ITS, LSU, and SSU except for *K.ebriosa* and *K.vinigena* using LSU (Rodríguez-Andrade et al. 2020), *K.esperanzae*, *K.guangdongensis*, *K.laojunensis*, and *K.ramus* using ITS and LSU (Raymundo et al. 2023; Senanayake et al. 2023; Zhang et al. 2023; Meng et al. 2024). In this study, we also conducted phylogenetic analyses using ITS, LSU, and SSU sequences, and our newly obtained ten strains nested within the genus *Kirschsteiniothelia* formed four independent lineages with reliable support value and can be recognized as three new phylogenetic species, namely *K.ganzhouensis*, *K.jiangxiensis*, and *K.jiulianshanensis*, and one known species, *K.inthanonensis*.

*Kirschsteiniothelia* is widely distributed in tropical and subtropical regions. Most species of this genus are known from dead woods or twigs in terrestrial and freshwater habitats, but occasionally, some species have been reported to be associated with orchid symbiosis, bioactive metabolites, and human infection (Poch et al. 1992; Nishi et al. 2018; Chen et al. 2022). Currently, with the addition of our species, there are 61 species in *Kirschsteiniothelia* (Tables 2–4), including 38 asexual species (Table 2), 18 sexual species (Table 3), and 5 species known for both morphs (Table 4), and 23 of which have dendryphiopsis-like asexual morphs and 20 have sporidesmium-like asexual morphs. In addition, on the basis of the typification, we found that members in *Kirschsteiniothelia* are mainly reported from China (22 species), Thailand (15 species), the USA (10 species), and India (4 species), whereas most regions are still essentially unrecorded. Thus, further research with the morpho-molecular approach is necessary to explore the hidden species diversity of *Kirschsteiniothelia* from different geographic regions and further focus on the correction of their asexual and sexual morphs, which will make significant contributions to the taxonomy of this genus and be necessary to quantify their roles in natural ecosystems.

**Table 2.** Synopsis of morphological characteristics, sequence data, type of asexual morph, habitat, host, and locality compared across *Kirschsteiniothelia* species with asexual morphs.

Species	Conidiophores (μm)	Conidiogenous cells	Conida		Sequence data	Type of asexual morph	Habitat/Host/ Locality	References
			Size (µm)	Morphology				
*Kirschsteiniotheliaacutispora*	180–260 × 7–12.5	Monoblastic	75–120 × 10.5–19.5	Obclavate to obspathulate, rostrate, mid to dark brown, becoming pale brown towards the apex, 7–12-euseptate	Present	Sporidesmium-like	Terrestrial/On dead branches/Thailand	Jayawardena et al. (2022)
*K.agumbensis*	9.05–14.95 × 7.25–8.5	Monoblastic	228–450.5 × 15–23.5	Cylindrical, rostrate, dark brown to brown, pale brown at apex, coarsely verrucose to granulate to punctate, 18–41-euseptate	Present	Sporidesmium-like	Terrestrial/On decaying wood of *Garcinia* sp./India	Sruthi et al. (2024)
*K.aquatica*	114–151 × 7–8	Monoblastic	35–46 × 7.5–8.5	Obclavate, subhyaline, dark brown at base, septate, sometimes percurrently proliferate at broken ends	Present	Sporidesmium-like	Freshwater/On submerged wood/China	Bao et al. (2018)
*K.arbuscula*	240–580 × 10–13	Monotretic	42–64 × 12–14	Subfusiform, fusiform, or obclavate, blackish olivaceous, 3–7 septate	Absent	Dendryphiopsis-like	Terrestrial/On bark of many plants/USA	Ellis (1976); Pratibha et al. (2010); Sruthi et al. (2024)
*K.binsarensis*	280–520 × 6.5–8	Monotretic	36–44 × 8–10	Obclavate to obclavate-fusiform, brown, 4–5-septate	Absent	Dendryphiopsis-like	Terrestrial/On dead branches/India	Subramanian and Srivastva (1994); Sruthi et al. (2024)
*K.biseptata*	Up to 180 × 8–10	Monotretic	28–39 × 19–22	Ellipsoidal or obovate, brown, 2-septate	Absent	Dendryphiopsis-like	Terrestrial/On dead twig/South Africa	Morgan-Jones et al. (1983); Sruthi et al. (2024)
*K.bulbosapicalis*	(47–)58–128(–199) × 7.5–12.5(–16.5)	Monoblastic	118–236.5 × 15–27	Cylindrical, ovoid to obclavate, rostrate, olivaceous to reddish-brown to dark brown, 8–13-septate, with a spherical hyaline mucilaginous sheath	Present	Sporidesmium-like	Terrestrial/On unidentified decaying wood/China	Tang et al. (2024)
*K.cangshanensis*	105.5–135.5 × 6–8	Monoblastic	33–43 × 7.5–8.5	Obclavate, pale brown to brown, with a gelatinous sheath at apex, septate	Present	Sporidesmium-like	Freshwater/On submerged wood/China	Bao et al. (2018)
*K.crustacea*	60–170 × 6.5–10.5	Monoblastic	45–75 × 10–18	Obclavate to obspathulate, rostrate, mid to dark brown and hyaline to light brown towards the apex, 5–6-euseptate	Present	Sporidesmium-like	Freshwater/On decaying bamboo/Thailand	Jayawardena et al. (2022)
*K.dendryphioides*	179–467 × 4.5–8	Monotretic	30–55 × 9–13.5	Cylindrical, oblong, and occasionally clavate, brown, 2–4-septate	Present	Dendryphiopsis-like	Freshwater/On decaying wood/China	Tang et al. (2024)
*K.dushanensis*	160–307 × 6.5–13	Monoblastic	62–81 × 12.5–18	Fusiform lower part and euseptate, narrower cylindrical upper part, rostrate, olivaceous brown to soot brown, pale brown or subhyaline at the apex, truncate and darkened at the base, sometimes with a mucilaginous sheath surrounding the tail-like upper part or the apex, 5–11-septate, with distoseptate	Present	Sporidesmium-like	Freshwater/On submerged wood/China	Yang et al. (2023)
*K.ebriosa*	40–150 × 4	Mono- to polytretic	8–14 × 4–5	Cylindrical with rounded ends, mostly catenate, brown to dark brown, 1–2(–5)-septate	Present	Dendryphiopsis-like	Freshwater/From sparkling wine/Spain	Rodríguez-Andrade et al. (2020)
*K.extensa*	80–230 × 6.5–9.5	Monoblastic	45–120 × 5–12	Obclavate, becoming pale brown to pale towards the apex, 5–8 euseptate	Present	Sporidesmium-like	Terrestrial/On decaying wood/Thailand	Jayawardena et al. (2022)
*K.fascicularis*	200–450 × 9–11	Monotretic	48–90 × 5–10	Long-clavate, strongly attenuated at the base, 3–8 septate	Absent	Dendryphiopsis-like	Terrestrial/On bark of *Liquidambar*/USA	Hughes (1958); Sruthi et al. (2024)
*K.fluminicola*	209–286 × 7–9	Monoblastic	47.5–86.5 × 8–10	Solitary to short-catenate, obclavate, rostrate, subhyaline to dark brown, with conspicuous, spherical guttules in almost all cells, multi-septate	Present	Sporidesmium-like	Freshwater/ Unidentified decaying wood/China	Bao et al. (2018)
*K.ganzhouensis*	146.8–200 × 7.1–10.1	Monotretic	20.3–65.8(–164) × 3.0–5.3	Obclavate, rostrate, subhyaline to brown, pale at apex, 2–7(–14)-distoseptate	Present	Dendryphiopsis-like	Terrestrial/On decaying wood/China	This study
*K.guangdongensis*	250–350 × 10–18	Monoblastic	290–300 × 42–50	Elongated, flask-shaped, blackish brown to black, apical cell paler than others, with a thin sheath at apex, 13-septate with one longitudinal septum in 5 basal cells	Present	Sporidesmium-like	Terrestrial/On plant twigs/China	Senanayake et al. (2023)
*K.goaensis*	85–230 × 4–6	Monotretic	20–40 × 5–7.5	Cylindrical, rounded at both ends, dark brown, 3–5-septate	Absent	Dendryphiopsis-like	Terrestrial/On dead and decaying bark/India	Pratibha et al. (2010)
*K.inthanonensis*	611–1549 × 2.5–6.6	Mono- to polytretic	24–230 × 5.7–14.3	Obclavate, rostrate, grey to brown, pale at apex, 2–10-euseptate	Present	Dendryphiopsis-like	Terrestrial/On twigs of *Quercusoleoides* /Thailand	de Farias et al. (2024)
*K.jiangxiensis*	32.9–90.4 × 7.3–12.9	Monoblastic	75.9–103.8 × 8.9–15.2	Obclavate, rostrate, pale brown to brown, 7–10-euseptate	Present	Sporidesmium-like	Terrestrial/On decaying wood/China	This study
*K.jiulianshanensis*	128.6–291.4(–430) × 7.1–10	Monotretic	31.4–57.1 × 10–11.4	Obclavate, rostrate, subhyaline to dark brown, 4–7-euseptate	Present	Sporidesmium-like	Terrestrial/On decaying wood/China	This study
*K.longirostrata*	80–252 × 4.5–9.5	Monoblastic	36.5–109(–160) × 8–16	Cylindrical, obpyriform to obclavate, rostrate, guttulate, 6–18-septate, proliferating, pale brown to brown, with a mucilaginous sheath surrounding the upper part of the apex	Present	Sporidesmium-like	Freshwater/On decaying wood/China	Tang et al. (2024)
*K.longisporum*	115–285 × 6.5–14	Holoblastic	35–130 × 8.5–15	Cylindrical-obclavate, elongated, brown, 3–15-distoseptate, verruculose	Present	Dendryphiopsis-like	Terrestrial/On dead branches of *Pinustaeda*/China	Tian et al. (2024)
*K.nabanheensis*	320–588 × 8–12	Monotretic	32–112 × 8–12	Obclavate or fusiform, sometimes rostrate, dark brown to brown, 3–7 euseptate	Present	Dendryphiopsis-like	Terrestrial/On dead branches/China	Liu et al. (2023)
*K.pini*	69–124 × 3.5–7	Monoblastic	22–45 × 5–10	Obclavate, becoming brown to pale towards the apex, 3–6-euseptate	Present	Dendryphiopsis-like	Terrestrial/On decaying branches of *Pinus*/China	Jin et al. (2024)
*K.puerensis*	100–250 × 5–12	Monoblastic	60–140 × 5–20	Obclavate, pale-brown to brown, pale-brown at the apex, sometimes with 1–2 hyaline sheaths around the tip, 5–12-septate	Present	Sporidesmium-like	Terrestrial/On dead wood of *Coffea*/China	Hyde et al. (2023)
*K.ramus*	102–248 × 5–11	Monotretic	42–56 × 15–22	Cylindrical, pale olivaceous when young, brown when mature, 2–3-septate, verruculose	Present	Dendryphiopsis-like	Freshwater/On decaying wood/China	Zhang et al. (2023)
*K.rostrata*	190–450 × 9–15	Monoblastic	80–150 × 10–20	Obclavate, rostrate, olivaceous brown to brown, pale at apex, sometimes with a mucilaginous sheath at apex, 8–13-septate	Present	Sporidesmium-like	Freshwater/On decaying wood/Thailand	Hyde et al. (2017)
*K.septemseptata*	250–580 × 6.5–14.5	Mono- to polytretic	25–55 × 6.5–12.5	Obclavate, rostrate, olivaceous brown to brown, pale at apex, 5–8 euseptate	Present	Dendryphiopsis-like	Terrestrial/On decaying wood/Thailand	Jayawardena et al. (2022)
*K.shimlaensis*	110–268 × 12–19	Monotretic	41–81 × 13–17.5	Obovoid, oblong, broad clavate or cylindrical, dark brown or black, microguttulate, lumen aspect granulose, 2–5(–6) septate	Absent	Dendryphiopsis-like	Terrestrial/On decaying stump of *Cedrusdeodara*/India	Verma et al. (2021)
*K.sichuanensis*	82–194 × 5–10	Monoblastic	34–54 × 8–14	Obclavate, becoming brown to pale towards the apex, 2–7 euseptate	Present	Dendryphiopsis-like	Terrestrial/On dead wood/China	Jin et al. (2024)
*K.spatiosa*	70–128 × 7.5–12.5	Monoblastic	90–139 × 9.5–16.5	Obclavate, rostrate, olivaceous brown to brown, pale at apex, sometimes with a mucilaginous sheath at apex, 8–23-euseptate	Present	Sporidesmium-like	Terrestrial/On decaying wood/Thailand	Jayawardena et al. (2022)
*K.submersa*	220–280 × 6–7	Monoblastic	37.5–51.5 × 8.5–9.5	Obclavate, brown to pale brown, hyaline and thinner at the tip, 4–6-septate	Present	Sporidesmium-like	Freshwaterl/On decaying wood/China	Su et al. (2016)
*K.tectonae*	Up to 200 × 4–8	Monoblastic	135–150 × 16–17	Cylindric-obclavate, elongate, dark reddish brown, with sheath at apex, 9–25 or more septa	Present	Sporidesmium-like	Terrestrial/On dead bark of *Tectonagrandis*/Thailand	Li et al. (2016)
*K.thailandica*	55–93 × 7–10	Monoblastic	74–110 × 13–20	Obclavate, olivaceous or brown, hyaline at apex, with a conspicuous, gelatinous, hyaline sheath around tip, 6–8-distoseptate	Present	Sporidesmium-like	Terrestrial/On twigs of *Ficusmicrocarpa*/ Thailand	Sun et al. (2021)
*K.vinigena*	100–150 × 3	Mono- to polytretic	8–80 × 4–5	Solitary or catenate, cylindrical, dark brown, smooth-walled to coarsely verrucose, 1–2(–7)-septate	Present	Dendryphiopsis-like	Terrestrial/ From cork stopper / Spain	Rodríguez-Andrade et al. (2020)
*K.xishuangbannaensis*	35–150 × 5–15	Monoblastic	30–150 × 5–20	Obclavate, rostrate, yellow-brown to brown, subhyaline or pale-brown at apex, some have guttules, 1–2 hyaline globose to ampulliform, mucilaginous sheaths around the tip, 3–8-septate	Present	Sporidesmium-like	Terrestrial/On dead branches of *Heveabrasiliensis*/China	Xu et al. (2023)
*K.zizyphifolii*	287–444.5 × 10.3–17(–19.7)	Tretic	37.6–46.5 × 13.5–19	Cylindrical to rarely clavate, brown dark to brown, 2–3-septate	Present	Dendryphiopsis-like	Terrestrial/On dead wood of *Nayariophytonzizyphifolium*/Thailand	de Farias et al. (2024)

Notes: All conidia are solitary and smooth except where indicated.

**Table 3.** Synopsis of morphological characteristics, sequence data, habitat, host, and locality compared across *Kirschsteiniothelia* species with sexual morphs.

﻿Species	Asci (μm)	Ascospores		Sequence data	Habitat/Host/Locality	References
		Size (μm)	Characteristics			
*Kirschsteiniotheliaabietina*	100–110 × 20	23–28 × 6–10	Irregularly biseriate, elliptical, rounded at the ends, slightly or not at all constricted at the septum, young hyaline, nucleosomes or granular, becoming brown	Absent	Terrestrial/On bark of *Tsugacanadensis*/USA	Wang et al. (2004)
*K.acerina*	85–95 × 20–24	22–26 × 8–11	Ellipsoid, 1-septate, the lower cell often somewhat smaller, slightly constricted at the septum, brown to dark brown, verruculose	Absent	Terrestrial/On absorbing mycorrhizal rootlets of *Acersaccharum*/USA	Hawksworth (1985)
*K.arasbaranica*	120–180 × 30–40	(30–)34–42(–44) × (12–)13–16(–18)	Narrowly to broadly ellipsoidal with rounded apex, brown to dark brown at maturity, verrucose to finely spinulose, covered with a mucilaginous sheath, guttulate, 1-septate, septum deeply constricted and submedian, the upper cell distinctly larger than the lower cell	Present	Terrestrial/On dead branch of *Quercuspetraea*/Iran	Mehrabi et al. (2017)
*K.atkinsonii*	70–90 × 9–16	14–16 × 5–6	2-seriate, clavate, light brown, upper cell wider than elongated lower cell, verrucose, 1-septate	Absent	Terrestrial/On leaves of *Freycinetiaarnotti*/USA	Hyde (1997)
*K.chiangmaiensis*	76–119 × 24–30	20–31 × 9–12	Bi- or tri-seriate in the middle and uniseriate in the top of the ascus, ellipsoid to fusiform, narrowly to broadly ellipsoidal with rounded or slightly pointed at the ends, 1-septate, septum submedian and deeply constricted, the upper cell distinctly larger than the lower cell, guttulate, brown, smooth, with a mucilaginous sheath	Present	Freshwater/On decaying wood/ Thailand	Louangphan et al. (2024)
*K.dolioloides*	125–145 × 25–34	84–39 × 14–15	2-celled, with a slight constriction at the septum, young olivine, old dark brown	Absent	Terrestrial/On bark of *Pinus*/Switzerland	Wang et al. (2004)
*K.esperanzae*	(168–)178–203 × 32–35	40–50(–53) × 14–17	Ellipsoid or soleiform, 1-septate, slightly constricted at the septum, light brown to olive-brown, smooth	Present	Terrestrial/On decaying wood/Mexico	Raymundo et al. (2023)
*K.laojunensis*	(105–)130–162(–180) × (17–)20–30	(34–)35–55(–56) × (10–)11–14(–16)	Fusiform, usually1-septate, slightly constricted at the septum, asymmetric with a slightly larger upper cell, both ends slightly subacute, guttulate, hyaline when young and turning dark brown with greenish or bluish coloration at maturity	Present	Terrestrial/On the bark of *Abiesfabri*/China	Meng et al. (2024)
*K.phileura*	–	22 × 10	Ellipsoid, 1-septate, the upper cell usually larger than the lower cell, somewhat constricted at the septum, brown	Absent	Terrestrial/On the bark of *Tiliaamerican*/USA	Barr (1993)
*K.phoenicis*	70–112 × 14–24	18–27 × 5–7.5	Ellipsoid, rounded or slightly pointed at the ends, brown, 1-septate, septum submedian and constricted, upper cell broader than the lower cell, guttulate, smooth, with a mucilaginous sheath	Present	Freshwater/On rachis of *Phoenixpaludosa*/Thailand	Hyde et al. (2018)
*K.populi*	80–90 × 8	12 × 6	Ovate, ends rounded, fuliginous, equally uniseptate, much constricted	Absent	Terrestrial/On decorticated branches of *Populusangustifolia*/USA	Wang et al. (2004)
*K.proteae*	54–72 × 6–8	(11–)13–17(–20) × 3–4(–5)	Fusoid, 1-septate, median or submedian, smooth, with germ pore at ascospore ends, at times cells become biguttulate, pale brown to brown	Absent	Terrestrial/On decorticated twig litter of *Proteacynaroides*/South Africa	Marincowitz et al. (2008)
*K.reticulata*	90–110 × 23–27	17–23 × 7–10	Ellipsoid, dark brown, 1-septate, septum constricted, median or occasionally submedian, with reticulate ornamentation on surface, covered with mucilaginous sheath	Absent	Terrestrial/On dead twigs/China	Chen et al. (2006)
*K.smilacis*	75–100 × 16–21	20–24 × 6–8	Ellipsoid, pale brown, 1-septate, slightly constricted at septum, wall finely and inconspicuously verrucose, covered with mucilaginous sheath	Absent	Terrestrial/On stem of *Smilax* sp./China	Chen et al. (2006)
*K.striatispora*	65–75 × 9–11	(14–)15–18(–19) × 5–6.5	Ellipsoid to somewhat soleiform, 1-septate, the cells equal in size or the lower slightly smaller, apices rounded, reddish-brown, slightly granular at first but finally with up to five longitudinal or sinuate furrows	Absent	Terrestrial/On dead twigs of Juniperuscommunissubsp.nana/Switzerland	Hawksworth (1985)
*K.thujina*	100–140 × 17–22	(29–)36–50(–55) × (12–) 15–17(–19)	Elongate-ellipsoid, slightly attenuated towards the apices, 1-septate, dark brown, apparently smooth walled, often guttulate	Present	Terrestrial/On decaying wood of *Thujaoccidentalis*/USA	Hawksworth (1985); Zhang and Fournier (2015)
*K.umbrinoidea*	–	23–28 × 75–9	Oblong-fusiform, hyaline, two guttulate	Absent	Terrestrial/On bark of *Aesculushippocastanum*/Italy	Wang et al. (2004)
*K.xera*	70–75 × –	17–23 × 6–7	1-septate, constricted at the septum, cells somewhat unequal, guttulate, with granulate contents, uniseriate or partly biseriate	Absent	Terrestrial/On bark of *Prunus*/USA	Wang et al. (2004)

Notes: All ascospores are smooth except where indicated.

**Table 4.** Synopsis of morphological characteristics, sequence data, type of asexual morph, habitat, host, and locality compared across *Kirschsteiniothelia* species with asexual and sexual morphs.

Species	Teleomorph		Anamorph		Sequence Data	Habitat/Host/Locality	References
	Asci (μm)	Ascosporous morphology	Morphology	Type of asexual morph			
*Kirschsteiniotheliaatra*	70–90 × 12–15	25–33 × 8.5–12 μm, ellipsoidal, rounded or somewhat constricted at the apices, 1-septate, the upper cell usually larger in size, somewhat constricted at the septum, brown, sometimes appearing almost smooth	Conidiophores 245–355 × 8–10 μm; Conidiogenous cells tretic, integrated, sometimes percurrent, terminal, becoming intercalary, new cell developing from apical or subapical part of subtending cell; Conidia 54–63 × 14–18 μm, solitary, cylindrical, rounded at the apex, and narrowly truncate at the base, brown, 3–4-septate	Dendryphiopsis-like	Present	Terrestrial/On dead wood/Czech Republic	Hawksworth (1985); Wijayawardene et al. (2014); Su et al. (2016)
*K.emarceis*	88–140 × 18–24	25–28 × 8–9 µm, biseriate, ellipsoidal, septum median to supra-median, dull green, becoming brown to dark brown at maturity, 1-septate, smooth	Conidiophores 162–271 × 7–14 μm; Conidia 45–56 × 14–15 µm, oblong to clavate, grayish, brown to dark brown, 3–4(–5)-septate, constricted at septa, smooth	Dendryphiopsis-like	Present	Terrestrial/On dead wood/Thailand	Boonmee et al. (2012)
*K.lignicola*	107–163 × 19–28.5	27–30 × 10–12 µm, biseriate, ellipsoidal, 1(–2) septate, with median septum or in lower part, some ascospores with secondary septum, dull green, brown to dark brown at maturity	Conidiophores 287–406 × 11–13 μm; Conidia 39–48 × 21–25 μm, obovoid to broadly, 1–2-septate, constricted at septa, smooth, dark brown, rounded at ends	Dendryphiopsis-like	Present	Terrestrial/On decaying wood/Thailand	Boonmee et al. (2012)
*K.recessa*	90 × 10	15–17.5 × 5–6.5 μm, elongate-ellipsoid, rounded at the apices, 1-septate, cells equal in size or the lower slightly smaller, slightly constricted at septum, pale brown, almost smooth or with a very weak verruculose ornamentation	Conidiophores 3.5–4.0 μm wide, red–brown, thick-walled, septate	Dendryphiopsis-like	Absent	Terrestrial/On rotten wood/USA	Hawksworth (1985)
*K.saprophytica*	8–125 × 18–23	13–25(–40) × 7–11(–14) µm, ellipsoid, upper cell broader than lower cell, pale brown to dark brown, 1-septate, guttulate, smooth	Conidiophores 90–216 × 8–12 μm; Conidiogenous cells monoblastic, terminal, cylindrical, brown to dark brown; Conidia 36–69 × 19–35 μm, cylindrical rounded at ends, 2–3 septa, dark brown to black, smooth	Dendryphiopsis-like	Present	Terrestrial/On dead wood/Thailand	de Farias et al. (2024)

## Supplementary Material

XML Treatment for Kirschsteiniothelia ganzhouensis

XML Treatment for Kirschsteiniothelia inthanonensis

XML Treatment for Kirschsteiniothelia jiangxiensis

XML Treatment for Kirschsteiniothelia jiulianshanensis

## ﻿Additional information

### Author ORCIDs

Ming-Gen Liao https://orcid.org/0009-0001-9537-1773

Xiu-Guo Zhang https://orcid.org/0000-0001-9733-8494

Zhao-Huan Xu https://orcid.org/0009-0008-2641-7783

Jian Ma https://orcid.org/0000-0001-9783-1860

### Data availability

All of the data that support the findings of this study are available in the main text or Supplementary Information.

## Supplementary materials

Supplementary material 1 The concatenated ITS, LSU and SSU sequences Data type fas https://binary.pensoft.net/file/1230880This dataset is made available under the Open Database License (http://opendatacommons.org/licenses/odbl/1.0/). The Open Database License (ODbL) is a license agreement intended to allow users to freely share, modify, and use this Dataset while maintaining this same freedom for others, provided that the original source and author(s) are credited.Xing-Xing Luo, Ming-Gen Liao, Ya-Fen Hu, Xiu-Guo Zhang, Zhao-Huan Xu, Jian Ma
